# All Hands on Deck: A Case Report of an Interdisciplinary Team Preventing Elder Financial Abuse at a Skilled Nursing Facility

**DOI:** 10.7759/cureus.99047

**Published:** 2025-12-12

**Authors:** Grace Yi, Nicholas S Cho, Karen Galvez-Maquindang, Christine Sun, Navid Darouian

**Affiliations:** 1 Internal Medicine, University of California Los Angeles (UCLA) David Geffen School of Medicine, Los Angeles, USA; 2 Medical Scientist Training Program, University of California Los Angeles (UCLA) David Geffen School of Medicine, Los Angeles, USA

**Keywords:** case report, elder financial abuse, interdisciplinary teams, prevention, skilled nursing facility

## Abstract

Elder financial abuse (EFA) is a common but under-reported form of maltreatment among elderly individuals. This case describes an 84-year-old woman with cognitive deficits residing in a skilled nursing facility (SNF), who was exploited by a purported friend who took funds, important documents, and attempted to designate herself as the patient’s power of attorney. The situation was quickly identified by the multidisciplinary care team at the SNF and mitigated through early action and collaboration. The case emphasizes the importance of multidisciplinary efforts to identify at-risk patients and the establishment of protocols surrounding abuse identification and reporting to protect elderly patients from financial exploitation and preserve their autonomy, dignity, and quality of life.

## Introduction

The elderly comprise a vulnerable patient population that faces unique medical and social comorbidities. With increases in global life expectancy in the past few decades, the proportion of elderly patients accessing healthcare services likewise continues to grow [1]. In addition to navigating complex medical comorbidities, which affect over half the elderly population [2], older patients experience functional and cognitive decline with age and often require assistance with basic needs, contributing to heightened risk of social vulnerability and exploitation [3].

Although elder financial abuse (EFA) is one of the most common forms of elder abuse, it remains critically under-reported [4]. In the United States, the prevalence of EFA is estimated to be around 36.9%, and financial exploitation costs the senior population almost 17 billion dollars per year [5]. Seniors who experience EFA frequently incur financial losses that lead to an inability to afford food, medications, or healthcare services, in addition to social losses such as isolation, decreased trust and quality of life, and increased reliance on others for basic needs. Heterogeneous definitions of EFA that vary by local and state jurisdictions pose challenges to developing streamlined and standardized EFA assessment tools. Even more, reporting a single incident of EFA often entails a laborious process involving multiple entities, including local police, Adult Protective Services, and financial institutions; for each reported case, as many as 24 cases are thought to go unreported [4].

Healthcare providers may be uniquely situated to identify and intervene on concerns of potential exploitation of elders. In this case report, we describe how a multidisciplinary care team expediently identified EFA of an elderly patient residing at a skilled nursing facility (SNF) and intervened appropriately to recover her assets and protect her from future financial exploitation.

## Case presentation

Background

An 84-year-old female was brought to the emergency room by ambulance after being found down at home by her home health wound care nurse. On arrival, the patient presented with aphasia, left-sided hemiparesis, and right-sided gaze deficit, and was not following commands. The wound care nurse reported that the patient was typically talkative and ambulatory and had been at her baseline two days prior. The patient’s last known well time was approximately 24 hours prior when she had attended an outpatient otolaryngology appointment for hearing loss, where she was accompanied by a friend who reportedly stated she would stop assisting the patient unless she pursued hearing aids. Of note, the patient lived alone, with all her family living in Scotland and Ireland.

**Figure 1:**
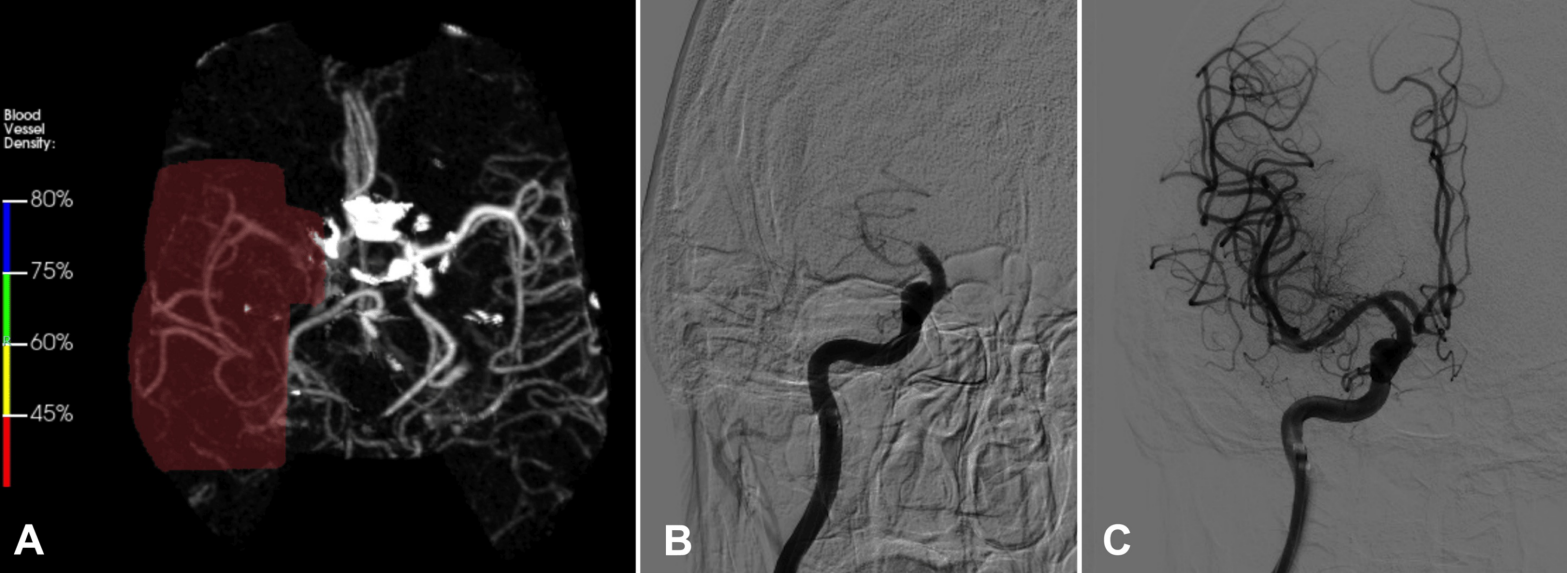
Representative cerebrovascular imaging scans of the patient. (A) Initial CT angiogram showing occlusion of the right M1 segment, (B) cerebral angiography performed pre-mechanical thrombectomy and (C) successful reperfusion post-mechanical thrombectomy

In the emergency room, her National Institutes of Health (NIH) Stroke Scale score was 27. A computed tomography angiogram (CTA) revealed a large vessel occlusion of the right M1 segment with reduced perfusion in the right middle cerebral artery territory (Figure 1A). Given the emergent situation with no family or surrogate medical decision-maker able to be contacted, informed consent could not be obtained, and the patient underwent emergent mechanical thrombectomy (Figures 1B-1C). Following the procedure, her NIH Stroke Scale score improved to 6. Post-operative magnetic resonance imaging (MRI) of the brain revealed multiple subacute infarcts along with chronic ischemic changes.

During her hospitalization, it became evident that the patient lacked a surrogate medical decision-maker. She expressed uncertainty in whom to designate, but she listed her friend from the otolaryngology appointment as her emergency contact. Upon discharge, she was motivated to continue physical therapy and was planned to go to an acute rehabilitation facility. However, due to insurance constraints, she was discharged to a skilled nursing facility.

Interdisciplinary team involvement

**Figure 2:**
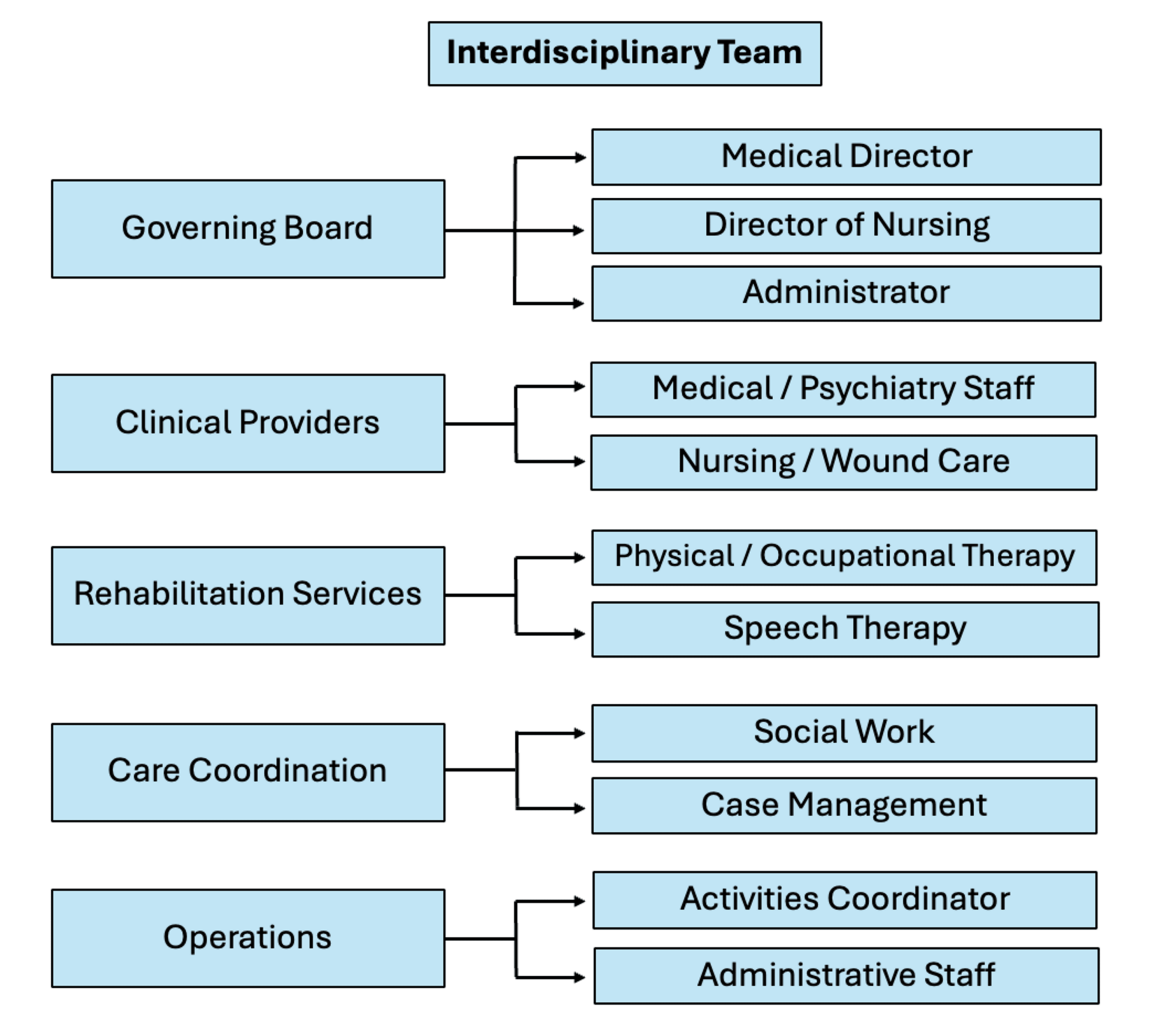
Overview of interdisciplinary team members at a skilled nursing facility

During the patient’s admission to the SNF for stroke rehabilitation, her interdisciplinary care team, including physical and occupational therapy, the director of nursing, social work, medical students, and the attending physician, raised concerns about her memory and ability to take care of herself. She frequently forgot recent health issues, including her pressure ulcer that prompted her receiving home health wound care and a recent urinary tract infection, and required frequent redirection. These findings prompted the interdisciplinary team (Figure 2) to request a formal neurocognitive evaluation to assess the patient’s medical decision-making capacity.

The patient was also visited by her friend on multiple occasions. The SNF staff recognized her friend, as she had been seen visiting other patients in the past. While initially the staff perceived her friend to be supportive, the friend’s behavior became concerning after the social worker identified unusual financial transactions between her and the patient. The friend also began taking the patient out of the facility for meals together. After returning from one such outing together, the staff noted that the friend was acting suspiciously and concealing items in her jacket. The social services director of the facility was contacted, and the friend demanded that the patient be transferred to a different SNF, claiming to be dissatisfied with the current SNF’s staff. As the director continued to question the friend, a search of her jacket revealed approximately $1,500 of the patient’s cash, which the friend claimed was their “lunch fund.” The SNF staff also found the patient’s social security card, California state identification card, and Medicare card in the friend’s jacket. Local law enforcement, adult protective services, the director of nursing, the attending physician, and facility administration were immediately notified.

Outcome and follow-up

The patient’s personal belongings and funds were successfully recovered by the SNF and secured in the facility. Further investigation revealed that the friend was attempting to designate herself as the patient’s durable power of attorney without legal authority. Following a formal neurocognitive evaluation by the SNF’s psychiatrist, the interdisciplinary care team initiated public guardianship proceedings through case management to safeguard the patient from further potential financial exploitation.

## Discussion

This report describes a case where EFA of an elderly patient residing at an SNF was identified and expediently addressed by a multidisciplinary care team. In the setting of residential long-term care settings such as SNFs, where staff have longitudinal interactions with patients and their visitors, care providers are well-positioned to identify concerns of EFA or repeated patterns warranting investigation [5]. This case underscores the importance and potential of interdisciplinary collaboration to recognize and mitigate elder abuse.

In this instance, each member of the interdisciplinary care team played a pivotal role in characterizing the patient’s social vulnerability and high risk of exploitation. For example, the physical and occupational therapist and the attending physician conferred about the patient’s ongoing cognitive decline and frailty, while the facility staff observed her visitor’s atypical behaviors and the social worker identified unusual financial transactions. Multidisciplinary communication between onsite staff enabled proactive detection of the visitor’s behaviors that eventually led to early identification of EFA and immediate intervention with case management to pursue legal guardianship.

The principle of guardianship is to protect and care for individuals who cannot make decisions about themselves or their property, and public guardianship refers to the appointment of a public official to serve as a legal guardian for such individuals [6]. For this patient, who had not previously identified surrogate decision-makers, had no family within the United States (US), and whose severe cognitive impairments precluded her from making informed decisions about her health and finances, public guardianship reduced her risk of financial and social exploitation at the hands of acquaintances or others with ulterior motives. Given that this case occurred in the US, it is important to note that the medico-legal processes used here are relevant to US legal structures surrounding public guardianship and long-term care facilities and may not be generalizable to other non-US settings.

Despite this patient’s successful outcome, many more cases of EFA remain overlooked and unreported [7]. Systemic barriers to detection include inconsistent validation and implementation of standardized screening tools for EFA [8]. Measures such as the Older Adult Financial Exploitation Measure (OAFEM) are based on self-reported data and may not be appropriate for adults with cognitive difficulties who reside at full-time care facilities [9]. Continued efforts should be made to streamline and implement screening tools for EFA in clinical settings or longitudinal care facilities, particularly for patients who may not be able to communicate concerns or complete assessments that rely on self-reported screening tools.

Provider time constraints and lack of training on recognizing or reporting signs of elder abuse may also impair timely identification and mitigation [10]. Interdisciplinary education on identifying and reporting concerns of exploitation should be integrated into standard onboarding protocols for care facility employees and continuing medical education training for all providers. Clear protocols on how to document and report suspected instances of elder abuse should be established and widely disseminated to staff at care facilities with elderly patients [10].

## Conclusions

Elder financial abuse is a pervasive but underrecognized form of elder abuse that can threaten older individuals’ financial stability, social independence, and quality of life. This case describes how an elderly patient with cognitive difficulties residing at an SNF was exploited by a reported “friend.” Through a coordinated effort by staff at the SNF, who quickly identified the abuse and initiated protective measures, the patient avoided potentially severe financial and legal harm. As healthcare utilization by elderly patients continues to increase, interdisciplinary care teams play a critical role in early recognition and mitigation of elder financial abuse in longitudinal care settings. In addition, developing and implementing standardized screenings for EFA in combination with establishing institutional protocols and collaborative educational efforts surrounding elder abuse may safeguard elderly patients against exploitation and protect their health and interests.
